# Supplementary material for: Moderate nucleotide diversity in the Atlantic herring is associated with a low mutation rate
Source: eLife. 2017 Jun 30;6:e23907. doi: 10.7554/eLife.23907 (PMC5524536; doi:10.7554/eLife.23907)
Supplement: Supplementary file 1. — DOI: http://dx.doi.org/10.7554/eLife.23907.008 [file elife-23907-supp1.docx]

**Supplementary File 1**. Estimated population size of major stocks of Atlantic herring in the North East Atlantic Ocean including the Baltic Sea based on assessment of the abundance at the start of the existing time series by the International Council for the Exploration of the Sea (ICES) (<http://www.ices.dk>). We chose the start of the time series since some of these stocks have since then been reduced by intensive fishing. This is an underestimate of the total census populations of Atlantic herring since the stocks in the West Atlantic Ocean are not included.

| Stock | Year | Estimated population size |
| --- | --- | --- |
| North Sea | 1947 | 1.2 x 10^11^ |
| Celtic Sea | 1958 | 1.4 x 10^9^ |
| West Scotland/Ireland | 1957 | 4.3 x 10^9^ |
| Irish Sea | 1961 | 1.9 x 10^8^ |
| Norwegian Spring Spawning | 1988 | 8.6 x 10^11^ |
| Iceland Summer Spawning | 1987 | 2.3 x 10^7^ |
| Western Baltic Sea | 1991 | 1.4 x 10^10^ |
| Central Baltic | 1974 | 6.0 x 10^10^ |
| Gulf of Bothnia | 1980 | 9.2 x 10^9^ |
| Gulf of Riga | 1977 | 4.5 x 10^9^ |
| Total |  | 1.0 x 10^12^ |
