# Supplementary material for: Moderate nucleotide diversity in the Atlantic herring is associated with a low mutation rate
Source: eLife. 2017 Jun 30;6:e23907. doi: 10.7554/eLife.23907 (PMC5524536; doi:10.7554/eLife.23907)
Supplement: Supplementary file 2. — DOI: http://dx.doi.org/10.7554/eLife.23907.009 [file elife-23907-supp2.docx]

| **Supplementary File 2. Summary statistics of the SNP calls underlying the estimation of the false negative rate by means of simulation.**  **Atlantic herring family Progeny ID** | | | | | | | | | | | | | | | | | | | | | | |
| --- | --- | --- | --- | --- | --- | --- | --- | --- | --- | --- | --- | --- | --- | --- | --- | --- | --- | --- | --- | --- | --- | --- |
|  | | | | |  |  |  |  |  |  |  |  |  |  |  |  |  |  |  |  |  |  |
|  | **AA1** | **AA2** | | **AA3** | | | **AA4** | | | **AA5** | | | **AA6** | | | | **Average** | | | |  |  |
| **Total number of SNPs†** | 912 | 909 | | 896 | | | 916 | | | 904 | | | 914 | | | | 909 | | | |  |  |
| **Number of correctly called heterozygous SNPs** | 885 | 890 | | 882 | | | 894 | | | 881 | | | 887 | | | | 887 | | | |  |  |
| **%Correctly called heterozygous SNPs** | 97.0 | 97.9 | | 98.4 | | | 97.6 | | | 97.4 | | | 97.0 | | | | 97.5 | | | |  |  |
| **%False negative** | 2.9 | 2.1 | | 1.5 | | | 2.4 | | | 2.5 | | | 2.9 | | | | 2.4 | | | |  |  |
|  |  | | | |  | | |  | | |  | | |  | | | | |  |  | | |
| **Baltic herring family** | **Progeny ID** | | | | | | | | | | | | | | | | | |  |  | | |
|  |  |  |  |  |  |  |  |  |  |  |  |  |  |  |  |  |  |  |  |  |  |  |
|  | **BB1** | **BB2** | | **BB3** | | | **BB4** | | | **BB5** | | | **BB6** | | | | **Average** | | | |  |  |
| **Total number of SNPs†** | 921 | 910 | | 907 | | | 913 | | | 911 | | | 912 | | | | 912 | | | |  |  |
| **Number of correctly called heterozygous SNPs** | 892 | 887 | | 879 | | | 889 | | | 886 | | | 877 | | | | 885 | | | |  |  |
| **%Correctly called heterozygous SNPs** | 96.8 | 97.4 | | 96.9 | | | 97.3 | | | 97.2 | | | 96.1 | | | | 97.0 | | | |  |  |
| **%False negative** | 3.1 | 2.5 | | 3.0 | | | 2.6 | | | 2.7 | | | 3.8 | | | | 3.0 | | | |  |  |
|  |  | | | |  | | |  | | |  | | |  | | | | |  |  | | |
| **Total** |  | | | |  | | |  | | |  | | |  | | | | |  |  | | |
|  | **Average** | |  | | |  | | |  | | |  | | |  |  | | | | | |  |
| **Total number of SNPs†** | 910 | |  | | |  | | |  | | |  | | |  |  | | | | | |  |
| **Number of correctly called heterozygous SNPs** | 886 | |  | | |  | | |  | | |  | | |  |  | | | | | |  |
| **%Correctly called heterozygous SNPs** | 97.2 | |  | | |  | | |  | | |  | | |  |  | | | | | |  |
| **%False negative** | 2.7 | |  | | |  | | |  | | |  | | |  |  | | | | | |  |
|  |  | | | |  | | |  | | |  | | |  | | | | |  |  | | |
|  |  | | | |  | | |  | | |  | | |  | | | | |  |  | | |
|  | | | | | | | | | | | | | |  | | | | |  |  | | |

**† Total number of SNPs after filtering according to criteria used for real data (see methods).**
