# Supplementary material for: Moderate nucleotide diversity in the Atlantic herring is associated with a low mutation rate
Source: eLife. 2017 Jun 30;6:e23907. doi: 10.7554/eLife.23907 (PMC5524536; doi:10.7554/eLife.23907)
Supplement: Supplementary file 3. — DOI: http://dx.doi.org/10.7554/eLife.23907.010 [file elife-23907-supp3.docx]

**Supplementary file 2.** Estimated generation times for commercially fished herring stocks.

| **Year** | **North Sea** | **West Scotland/ Ireland** | **Irish Sea** | **Central Baltic** | **Norwegian Spring Spawning** | | **Celtic Sea** |
| --- | --- | --- | --- | --- | --- | --- | --- |
| 1947 | 6.34 | n/a | n/a | n/a | | n/a | n/a |
| 1948 | 6.20 | n/a | n/a | n/a | | n/a | n/a |
| 1949 | 5.76 | n/a | n/a | n/a | | n/a | n/a |
| 1950 | 5.45 | n/a | n/a | n/a | | n/a | n/a |
| 1951 | 5.49 | n/a | n/a | n/a | | n/a | n/a |
| 1952 | 5.34 | n/a | n/a | n/a | | n/a | n/a |
| 1953 | 5.24 | n/a | n/a | n/a | | n/a | n/a |
| 1954 | 5.16 | n/a | n/a | n/a | | n/a | n/a |
| 1955 | 4.92 | n/a | n/a | n/a | | n/a | n/a |
| 1956 | 5.05 | n/a | n/a | n/a | | n/a | n/a |
| 1957 | 5.11 | 4.69 | n/a | n/a | | n/a | n/a |
| 1958 | 5.34 | 4.93 | n/a | n/a | | n/a | 5.77 |
| 1959 | 4.35 | 5.03 | n/a | n/a | | n/a | 5.03 |
| 1960 | 4.82 | 4.70 | n/a | n/a | | n/a | 5.06 |
| 1961 | 4.98 | 4.87 | 5.28 | n/a | | n/a | 5.29 |
| 1962 | 5.71 | 5.17 | 5.37 | n/a | | n/a | 4.96 |
| 1963 | 4.41 | 5.11 | 5.46 | n/a | | n/a | 5.02 |
| 1964 | 4.75 | 5.06 | 4.88 | n/a | | n/a | 4.33 |
| 1965 | 4.99 | 5.46 | 4.16 | n/a | | n/a | 4.51 |
| 1966 | 4.75 | 4.47 | 4.27 | n/a | | n/a | 4.46 |
| 1967 | 4.82 | 4.84 | 4.09 | n/a | | n/a | 4.36 |
| 1968 | 4.60 | 5.17 | 3.87 | n/a | | n/a | 4.17 |
| 1969 | 3.91 | 5.44 | 4.05 | n/a | | n/a | 4.29 |
| 1970 | 3.67 | 5.30 | 4.11 | n/a | | n/a | 4.59 |
| 1971 | 3.76 | 5.19 | 4.00 | n/a | | n/a | 4.17 |
| 1972 | 3.61 | 4.53 | 4.19 | n/a | | n/a | 4.04 |
| 1973 | 3.76 | 4.68 | 4.27 | n/a | | n/a | 4.08 |
| 1974 | 3.94 | 5.23 | 3.83 | 4.65 | | n/a | 4.10 |
| 1975 | 4.09 | 5.28 | 3.84 | 4.70 | | n/a | 4.06 |
| 1976 | 3.51 | 4.83 | 4.02 | 4.98 | | n/a | 3.79 |
| 1977 | 3.98 | 4.79 | 4.04 | 5.00 | | n/a | 3.71 |
| 1978 | 4.01 | 4.79 | 3.89 | 4.87 | | n/a | 3.90 |
| 1979 | 4.11 | 4.51 | 3.91 | 4.86 | | n/a | 3.67 |
| 1980 | 4.33 | 4.54 | 3.89 | 5.19 | | n/a | 3.70 |
| 1981 | 4.13 | 4.76 | 3.78 | 5.15 | | n/a | 3.25 |
| 1982 | 4.04 | 4.73 | 3.83 | 4.66 | | n/a | 2.80 |
| 1983 | 3.96 | 4.88 | 4.01 | 4.35 | | n/a | 2.96 |
| 1984 | 3.85 | 4.28 | 4.31 | 4.45 | | n/a | 3.17 |
| 1985 | 4.10 | 4.43 | 4.50 | 4.37 | | n/a | 3.29 |
| 1986 | 4.25 | 4.62 | 4.43 | 4.29 | | n/a | 3.39 |
| 1987 | 4.09 | 4.94 | 4.69 | 4.40 | | n/a | 3.28 |
| 1988 | 4.03 | 4.73 | 4.46 | 4.38 | | 5.06 | 3.54 |
| 1989 | 4.30 | 4.86 | 4.61 | 4.60 | | 5.86 | 3.74 |
| 1990 | 4.69 | 5.26 | 4.62 | 4.53 | | 6.59 | 3.90 |
| 1991 | 4.99 | 5.59 | 5.09 | 4.13 | | 7.43 | 4.34 |
| 1992 | 5.37 | 5.95 | 4.78 | 4.16 | | 8.03 | 3.70 |
| 1993 | 5.64 | 5.44 | 5.06 | 3.96 | | 8.34 | 3.68 |
| 1994 | 4.90 | 5.70 | 4.64 | 4.22 | | 8.70 | 3.55 |
| 1995 | 4.59 | 5.97 | 4.44 | 4.10 | | 8.71 | 3.54 |
| 1996 | 4.54 | 5.39 | 4.70 | 3.99 | | 7.69 | 3.78 |
| 1997 | 4.53 | 5.26 | 4.58 | 3.89 | | 6.99 | 3.96 |
| 1998 | 4.49 | 4.59 | 4.15 | 4.13 | | 6.97 | 4.08 |
| 1999 | 4.57 | 4.72 | 4.16 | 4.17 | | 7.53 | 3.77 |
| 2000 | 4.88 | 5.11 | 4.26 | 4.43 | | 8.17 | 3.39 |
| 2001 | 4.33 | 4.68 | 4.54 | 3.98 | | 8.48 | 3.27 |
| 2002 | 4.46 | 4.73 | 4.45 | 3.94 | | 8.73 | 3.22 |
| 2003 | 4.59 | 4.90 | 4.11 | 4.12 | | 7.87 | 3.77 |
| 2004 | 4.83 | 5.29 | 3.73 | 3.92 | | 7.35 | 3.79 |
| 2005 | 5.16 | 5.26 | 3.67 | 4.28 | | 7.47 | 3.05 |
| 2006 | 5.43 | 5.74 | 3.68 | 4.34 | | 7.27 | 3.30 |
| 2007 | 5.54 | 5.51 | 3.60 | 4.21 | | 7.07 | 3.49 |
| 2008 | 5.41 | 6.03 | 3.75 | 4.25 | | 7.13 | 3.85 |
| 2009 | 5.26 | 6.33 | 4.00 | 3.87 | | 7.05 | 3.72 |
| 2010 | 5.63 | 6.23 | 4.01 | 3.98 | | 7.29 | 3.72 |
| 2011 | 5.15 | 5.84 | 4.17 | 4.09 | | 7.72 | 3.82 |
| 2012 | 4.94 | 5.06 | 4.35 | 4.60 | | 8.22 | 4.04 |
| 2013 | 5.20 | 5.71 | 4.45 | 4.40 | | 8.67 | 4.46 |
| 2014 | 5.09 | 6.17 | 4.64 | 4.45 | | 9.08 | 4.84 |

n/a: data not available
